# Fast, automated, continuous energy scans for experimental phasing at the BioMAX beamline

**DOI:** 10.1107/S1600577523005738

**Published:** 2023-08-01

**Authors:** Ishkhan Gorgisyan, Paul Bell, Michele Cascella, Mikel Eguiraun, Áureo Freitas, Julio Lidon-Simon, Jie Nan, Carla Takahashi, Hamed Tarawneh, Thomas Ursby, Ana Gonzalez

**Affiliations:** a MAX IV Laboratory, Fotongatan 2, 224 84 Lund, Sweden; Paul Scherrer Institute, Switzerland

**Keywords:** MX beamline, MAD SAD phasing, continuous energy scan, flying scan, motion synchronization

## Abstract

In X-ray macromolecular crystallography (MX), single-wavelength anomalous dispersion (SAD) and multi-wavelength anomalous dispersion (MAD) techniques are commonly used for obtaining experimental phases. For an MX synchrotron beamline to support SAD and MAD techniques it is a prerequisite to have a reliable, fast and well automated energy scan routine. This work reports on a continuous energy scan procedure newly implemented at the BioMAX MX beamline at MAX IV Laboratory. The continuous energy scan is fully automated, capable of measuring accurate fluorescence counts over the absorption edge of interest while minimizing the sample exposure to X-rays, and is about a factor of five faster compared with a conventional step scan previously operational at BioMAX. The implementation of the continuous energy scan facilitates the prompt access to the anomalous scattering data, required for the SAD and MAD experiments.

## Abstract 2

A novel continuous energy scan routine has been implemented at the BioMAX beamline at MAX IV Laboratory. It is about five times faster than the conventional step scanning routine and facilitates user experiments utilizing the single-wavelength anomalous dispersion and multi-wavelength anomalous dispersion techniques.

## 1. Introduction

Tunable synchrotron beamlines dedicated to macromolecular crystallography (MX) often have the capability to perform energy scans over the absorption edges of anomalous scatterers that can be present in the crystals. The measurement and analysis of the fluorescence counts allow the identification of the energies at which the real and imaginary components of the anomalous scattering factor *f*′ and *f*′′ reach local extreme values, which is important for phasing macromolecular structures by multi- or single-wavelength anomalous dispersion (MAD, SAD) (Guss *et al.*, 1988 ▸; Smith, 1991 ▸; Hendrickson & Ogata, 1997 ▸). Although molecular replacement is now the most widespread phasing method, aided by the breakthrough of *AlphaFold* model predictions (McCoy *et al.*, 2022 ▸), anomalous phasing remains the main method to determine macromolecular structures *de novo* and is required in the cases where a good prediction of the structure is not available. In addition, using fluorescence measurements over the absorption edge, it is possible to confirm the presence of metals in the samples. Furthermore, these kinds of measurements can also be used in functional studies of proteins to determine the oxidation state of metals in the spatially resolved anomalous dispersion (SpReAD) refinement (Einsle *et al.*, 2007 ▸; Lennartz *et al.*, 2022 ▸). Typically, the energy scans implemented on MX beamlines encompass a spectrum spanning about 100–200 eV. Measuring the fluorescence at 50–100 eV from the absorption edge serves to obtain a reasonable estimate of the anomalous scattering factor at the XANES (X-ray absorption near-edge structure) region of the absorption edge by normalizing the fluorescence counts to the theoretical values further below and above the edge (Evans & Pettifer, 2001 ▸). Usually MAD/SAD capable beamlines have an optical configuration which minimizes changes in the beam position over a relatively small energy range and facilitates fast collection of the scan without beam realignment. For example, the BioMAX beamline at MAX IV (Ursby *et al.*, 2020 ▸) can achieve near constant output with a double-crystal monochromator and a fast feedback system that acts on the second monochromator crystal (see Section 2.1). Fully automated X-ray fluorescence scans at BioMAX were first implemented as conventional step scans. In this operation mode, the sample would only be exposed to the X-ray beam for data acquisition once all the motors involved in the energy change had finished moving for each energy step. These step scans took about 2–3 minutes, not counting the duration of the scan preparation and recovery phases. The overall duration of the step scan was well in excess of the typical time (18–36 s) required to collect a full rotation data set at the beamline. This served as a motivation to improve the scanning procedure and reduce its overall duration.

Previously, efforts have been made to realize continuous scans in different types of beamlines applying various techniques (Joly *et al.*, 2014 ▸; Poswal *et al.*, 2016 ▸). In an MX beamline Stepanov *et al.* (2011 ▸) achieved continuous scans using only the motion of the monochromator. Here we describe the implementation of fast X-ray fluorescence scans at BioMAX, where a PandABox (Zhang *et al.*, 2017 ▸) is used to synchronize the motion of the monochromator and the undulator gap as well as the continuous 100 Hz data acquisition with an Xspress 3 Mini detector readout unit (Quantum Detectors, UK). With this system, the duration of the scan itself has been reduced by about two orders of magnitude, lasting as short as 1 s, while the overall duration of the scanning routine has become a factor of four or five shorter. Even though the continuous energy scan routine is described here for the BioMAX beamline, it is worth noting that the general concept can be of use for other (including non-MX) beamlines as well. Having a similar hardware and infrastructure (as described below), only small beamline/facility-specific modifications would be required to implement the routine elsewhere. An example of a similar technique applied at a soft X-ray spectroscopy beamline (FlexPES) at MAX IV is reported by Enquist *et al.* (2021 ▸).

## 2. The BioMAX beamline

The BioMAX beamline started operation in 2017 as the first MX beamline at MAX IV Laboratory. Thanks to the multi-bend achromat technology of the 3 GeV storage ring (Einfeld *et al.*, 2014 ▸) at MAX IV, BioMAX is able to provide users with a stable, tightly focused high-intensity X-ray beam with a photon flux over 10^13^ photons s^−1^ at a ring current of 400 mA. Being the first MX beamline at MAX IV, BioMAX was designed to support a wide array of MX experiments, including MAD and SAD phasing, and is easily tunable in the energy range 6–24 keV.

The beamline controls (Eguiraun *et al.*, 2017 ▸) are implemented using the Tango control system (Verdier *et al.*, 2011 ▸) and the *Sardana* software package (Coutinho *et al.*, 2011 ▸).

### 2.1. Beamline components

**Figure 1:**
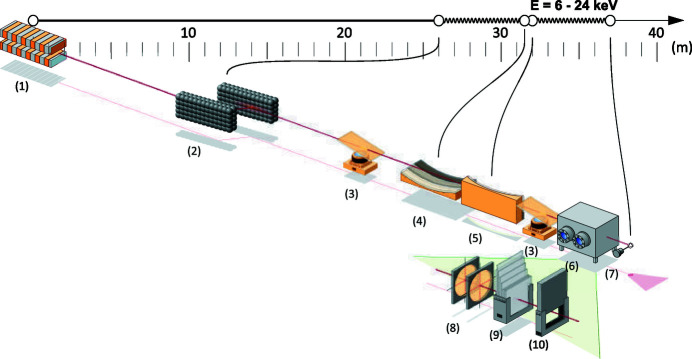
Main components of the BioMAX beamline: (1) Undulator, (2) horizontal double-crystal monochromator, (3) nanoBPMs, (4) vertically focusing mirror, (5) horizontally focusing mirror, (6) beam conditioning unit, (7) diamond XBPMs, (8) attenuators, (9) fast shutter.

A detailed description of the BioMAX beamline has been published by Ursby *et al.* (2020 ▸). This section covers mainly the beamline components relevant to the energy scan procedure. These components are illustrated in Fig. 1 ▸.

The X-ray beam of BioMAX is produced in a room-temperature in-vacuum undulator (Hitachi Metals, Japan) based on the technology by Yamamoto *et al.* (1992 ▸) that allows the generation of a high-brilliance photon beam.

**Figure 2:**
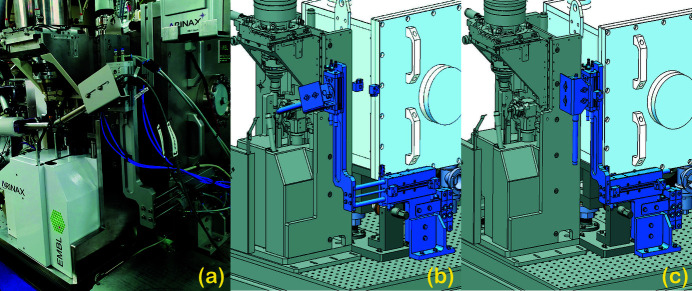
Fluorescence detector assembly photograph (*a*) in the *In* position and the model of the system in the *In* (*b*) and the *Out* (*c*) positions at the BioMAX endstation.

The next major component following the undulator is the horizontal double-crystal monochromator (HDCM) (FMB Oxford, UK) that utilizes two symmetrically cut Si (111) crystals, and allows the selection of the outgoing photon beam energy with a relative bandwidth of 2 × 10^−4^. Downstream of HDCM is located a NanoBPM (Van Silfhout *et al.*, 2011 ▸) (FMB Oxford, UK) that is used for photon beam position and transverse profile measurement. The measured positions are used as input to a 10 Hz feedback loop that controls the pitch and roll piezo motors of the HDCM second crystal in order to keep the beam at a predefined fixed position. The beam is focused using Kirkpatrick–Baez mirror geometry (Kirkpatrick & Baez, 1948 ▸) (Winlight X, France) that enables the X-ray beam transverse size (H × V) adjustment in a range from 20 µm × 5 µm to 100 µm × 100 µm for the routine operation. The surfaces of both the horizontally (HFM) and vertically (VFM) focusing mirrors have three stripes of different materials (Si, Rh, Pt) that are automatically selected depending on the operation energy of the beamline. After the focusing mirrors the X-ray beam passes through the beam conditioning unit (BCU) (Cianci *et al.*, 2017 ▸). Among different components in the BCU are two diamond X-ray beam position monitors (XBPMs) (CIVIDEC Instrumentation GmbH, Austria) that are used for measuring the transverse beam position and the intensity. The XBPM readings are fed to a 10 Hz PID feedback loop that controls the pitch of the HFM and VFM mirrors to keep the beam aligned to the sample position. Both feedback loops from NanoBPM and XBPM operate in parallel to compensate for possible drifts or fluctuations from the front-end or electron beam and the beamline optics, respectively. A set of three filter wheels is used in the BCU for adjusting the beam transmission. The BCU is followed by a Colibri fast shutter (Arinax, France) that can be controlled by an external open/close/gate signal. BioMAX is also equipped with an XR-100SDD fluorescence detector (Amptek, USA) which is used for X-ray fluorescence (XRF) measurements as well as for the energy scans. The detector is mounted on a pneumatic actuation system that allows its rapid positioning close to the sample during the measurements and retraction when not in use. Figure 2 ▸ shows the fluorescence detector along with the actuation system in the operation and parking positions. The BCU is also indicated in the drawings in light blue.

Data acquisition from the fluorescence detector is performed by an Xspress 3 Mini unit (Quantum Detectors, UK) that has a count rate of up to 4 Mcounts s^−1^ and a dead-time of around 80 ns. The device is able to measure the X-ray fluorescence in the energy range 0–40950 eV. It is also possible to specify a region of interest (RoI) in order to measure the counts only from a specific energy range. The data acquisition and exposure time can be controlled by an external signal using the *External Gate* mode of the device.

BioMAX utilizes a PandABox (Zhang *et al.*, 2017 ▸) for triggering of the Xspress 3 Mini and the Colibri shutter, as well as overall timing control of the energy scan routine.

### 2.2. Beamline energy

The energy of the beamline is controlled by a pseudomotor with a complex logic that simultaneously moves various motors to predefined positions corresponding to the set energy value. Besides the Bragg angle of the HDCM and the undulator gap, other motors involved in automated energy changes are the perpendicular distance and pitch of the HDCM second crystal and the KB mirror coating-selecting translation (only for work at the low- or high-energy regions of the beamline). The routine energy changes do not control the motion synchronization of the motors and require a settling time to ensure that all the individual motors have reached their final positions. As the motors do not move synchronously, the beam alignment is affected during the energy change, which is subsequently corrected by the two feedback loops described in Section 2.1. During the user operation, the energy change is followed by a routine called *checkbeam* that makes sure and, if needed, waits until the beam is aligned to the nominal position within predefined margins. Depending on the energy change, beam misalignment and the efficiency of the feedback loops, the correction (and therefore the wait) can take from several seconds up to a couple of minutes. The longest energy changes involve translation of the KB mirrors to use a different coated stripe.

A typical range of an energy scan is 100 eV, and for such a small energy change the perpendicular translation and pitch motors of the HDCM second crystal do not move, while the translation of the KB mirrors can be avoided during the scan procedure. As a result, only the motion of the Bragg angle and the undulator gap motors needs to be considered for the particular application of an energy scan.

**Figure 3:**
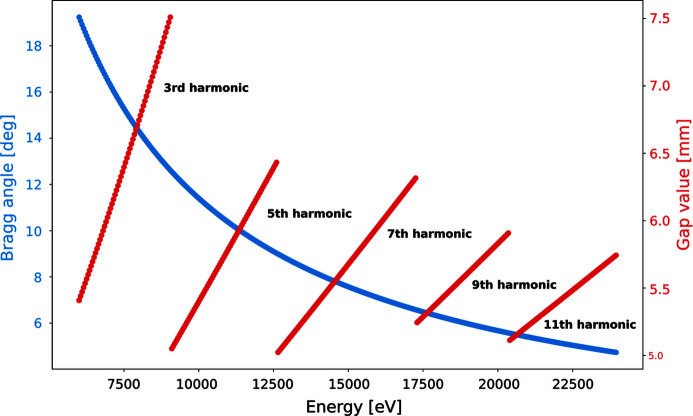
Bragg angle and undulator gap values for the total energy range of the BioMAX beamline.

The values of the Bragg angle and the undulator gap for different energies are shown in Fig. 3 ▸. One can see from the figure (red curves) that the odd harmonics 3rd to 11th of the undulator are used to cover the whole energy range of BioMAX.

Although the energy dependence is non-linear for both motors, as we will discuss in Section 4.2, for a short (100 eV) energy range a linear approximation can be used. By changing synchronously the undulator gap and the Bragg angle of the HDCM at a constant speed, we can continuously scan the X-ray beam energy while keeping the beam position and intensity stable.

## 3. Energy scan procedure

**Figure 4:**
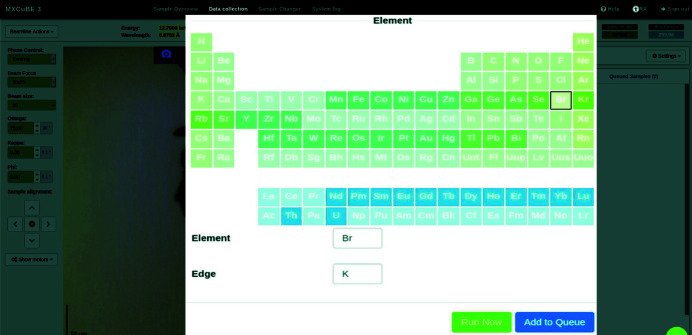
*MXCuBE3* interface for launching the energy scan.

The energy scan procedure is launched from *MXCuBE3* (Gabadinho *et al.*, 2010 ▸; Mueller *et al.*, 2017 ▸; Oscarsson *et al.*, 2019 ▸) which is the user interface for data collection at the BioMAX beamline. After centring the sample and creating a measurement point on it, the users are able to choose the element and the absorption edge they are interested in. This is done from a periodic table presented in the user interface (Fig. 4 ▸). Only the elements with *K* or *L* absorption edges within the beamline energy range can be selected in the interface. For *L* sub-edges, the one with lowest energy (usually *L*3) is used.

**Figure 5:**
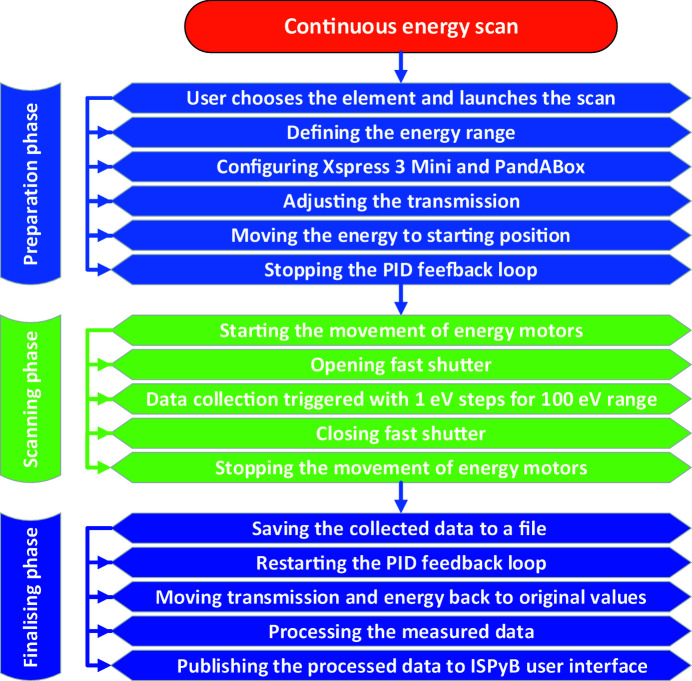
Flowchart of the energy scan procedure.

Once the element and absorption edge are chosen, the user can press the ‘Run now’ button to initiate the energy scan. This will continuously scan the X-ray beam energy while measuring the fluorescence emission from the element in question. The scan itself is as short as 1 s (excluding preparation and recovery phases) and covers an energy range of 100 eV with a total of 100 data acquisitions across the range. The resulting data are then analysed by a routine called *CHOOCH* (Evans & Pettifer, 2001 ▸) to retrieve the inflection, peak and remote energy values which are used for SAD and MAD data collections. The full procedure of energy scan is illustrated in the flowchart in Fig. 5 ▸. A more detailed description of different phases and individual steps shown in the flowchart is given in the sections below.

### 3.1. Preparation for the scan

During the preparation of the energy scan the fast shutter is kept closed at all times to avoid any unnecessary radiation on the crystal. The first step of the energy scan routine is to retrieve from a lookup table the energy value of the absorption edge and the emission (*K*_α_ or *L*_α_) energies for the chosen element. A 100 eV energy range is then defined around the absorption edge to perform the scan from *E*_edge_ − 50 eV to *E*_edge_ + 50 eV, where *E*_edge_ is the absorption edge energy. The program then checks if there is any harmonic jump (Fig. 3 ▸) in the defined scan range. If that is the case, the scan range is modified to exclude the harmonic jump. In the case when the harmonic jump happens closer than 30 eV from *E*_edge_, the scan is aborted with a relevant error message. Of all the heavy atoms commonly found or introduced in biological macromolecules, only Se has an absorption edge (*E*_Se_ = 12657.8 eV) that is less than 50 eV away from the jump between the fifth and seveth harmonics (*E*_H5→H7_ = 12610 eV): 30 eV < *E*_Se_ − *E*_H5→H7_ < 50 eV; thus in this case the scan is carried out between *E*_H5→H7_ + 1 eV and *E*_Se_ + 50 eV, that is from 12611 eV to 12707.8 eV. Having the emission energy for the chosen element, the preparation routine configures the Xspress 3 Mini, setting its RoI to a range *E*_em_ ± 150 eV, where *E*_em_ is the emission energy value. Such a RoI excludes signals from elastic scattering and possible emission from other electron shells or elements. This results in a lower background and higher sensitivity of the signal to the scanning energy.

The next step is to adjust and optimize the transmission of the X-ray beam. This is done to optimize the signal on the fluorescence detector without exceeding the count rate at which the signal is no longer linear. For this purpose, the X-ray beam energy is moved to a value slightly above the absorption edge (*E*_edge_ + 30 eV) to have an emission signal similar to the maximum of the energy scan. After the energy move the *checkbeam* (Section 2.2) routine is executed to make sure that the beam is well aligned. The transmission is then changed to a default value of 0.5%. This value is appropriate for the setup at BioMAX, based on a number of tests with different samples. At the default transmission a single measurement is performed to evaluate the counts and adjust the transmission accordingly. Before starting the measurement, the fluorescence detector is inserted in the position close to the sample [Fig. 2 ▸(*a*)]. The acquisition is done using a TTL gate signal that simultaneously opens the fast shutter to expose the sample to the X-ray beam, and triggers the Xspress 3 Mini acquisition unit. A 7 ms delay is applied to the gate signal of the detector with respect to that of the fast shutter in order to take into account the opening time of the latter and to ensure that the data are acquired while the shutter is fully open. The duration of the gate signal is set to 9 ms which is equivalent to the acquisition during a single step of the energy scan. If the number of counts from the single acquisition (*N*_0_) is within the defined limits (1000–5000), the default transmission of 0.5% remains unchanged; otherwise the transmission is multiplied by a factor 2500/*N*_0_ to linearly scale it for the desired number of counts of 2500. Such linear scaling is done only up to a maximum value of 10% to avoid excessive exposure of the sample to the X-ray beam. In the situation where *N*_0_ = 0, the transmission is not adjusted and a message is sent to the user as this could be caused by a misaligned beam or an incorrect acquisition.

Once the transmission value is defined for the scan, the energy of the X-ray beam is moved to the starting position of the scan *E*_edge_ − 50 eV, followed by a *checkbeam* routine to get the beam well aligned and stable for the start of the continuous energy scan. As the PID feedback loop described in Section 2.1 is too slow to correct possible misalignments of a 1 s scan (further discussed Section 4.3), it is disabled just before the start and is reactivated upon the completion of the scan.

### 3.2. Energy scan

**Figure 6:**
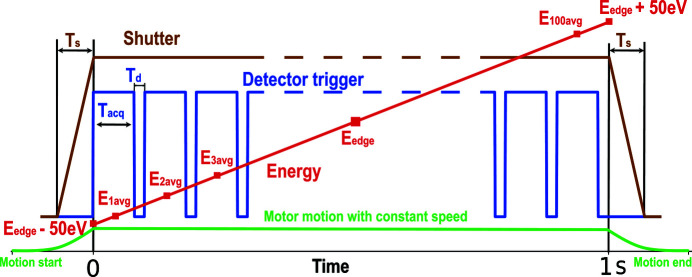
Timing illustration of energy scan: *T*_s_ is the opening/closing time of the fast shutter, *T*_acq_ and *T*_d_ are, respectively, acquisition and dead-times of the fluorescence detector. *E*_avg_ corresponds to the average energy value over a single acquisition.

The continuous energy scan executes synchronous motion of undulator gap and Bragg motors while simultaneously triggering the Xspress 3 Mini for fluorescence signal acquisition. The synchronization of the motors is achieved using a *Sardana* scanning routine (Fernández-Carreiras *et al.*, 2013 ▸), while the triggering logic and data acquisition is controlled by a PandABox. The scanning routine takes as input the starting and final positions of the undulator gap and Bragg motors as well as the number of acquisitions during the scan, the single acquisition duration and the dead-time. A timing sketch of the energy scan is illustrated in Fig. 6 ▸ where the energy is shown in red, the detector trigger in blue, the fast shutter in brown and the motor motion speed in green. The starting and final positions of the motors for *E*_edge_ ± 50 eV can be retrieved from the plots in Fig. 3 ▸. The number of acquisitions is set to 100 with exposure and dead-times of 9 ms and 1 ms (*T*_acq_ and *T*_d_ in Fig. 6 ▸), respectively. This amounts to a total duration of 1 s for the scan. Having the total duration and the movement range of the motors, the scanning routine calculates and adjusts their speeds before starting the motion. Using the acceleration values of the motors, a pre-starting position is calculated for each of them. This allows the undulator gap and Bragg motors to start the motion early and reach their respective nominal speeds by the time they arrive to the starting position of the scan. Similarly, the motion continues after having passed the final position of the scan allowing the motors to slow down and stop outside of the scanning range. This is indicated by the curved parts of the green line in Fig. 6 ▸.

Such motion ensures that both of the motors start their linear movement at the same time from position *E*_edge_ − 50 eV, move with a constant speed throughout the scanning range and simultaneously reach the final position *E*_edge_ + 50 eV. Given the small energy range, the linear change of the Bragg angle and the undulator gap can be considered equivalent to a linear change in energy with an average speed of 100 eV s^−1^. The linearity of the motion is further discussed in Section 4.2. It is worth noting that the scanning routine itself is not limited to the parameters (scanning range, number of acquisitions, total duration *etc*.) specified above. The only limiting factor is the speed of the physical motors to cover the required motion range within the defined time. The above-mentioned scanning parameters were chosen specifically for the current energy scan application. For 100 eV s^−1^ energy change the speeds of both undulator gap and Bragg motors are well within their physical limits for the entirety of the beamline energy range.

The PandABox is reading the encoder values of both motors at 1 MHz rate and knows their exact positions throughout the movement. As the Bragg angle is the one defining the final photon energy value at the sample location, PandABox uses its position for the triggering logic. Because the trajectory of the Bragg motor is well defined for each *E*_edge_ value, the PandABox creates a table of consecutive positions of the motor corresponding to 1 eV energy change and uses them to trigger the acquisition of the detector. Having a position-based triggering logic ensures that the program knows the exact energy value for each fluorescence signal acquisition, even if the motor motion is not perfectly linear in energy. The first checkpoint is the Bragg motor position corresponding to *E*_edge_ − 50 eV. A pre-trigger is sent to the fast shutter 7 ms before this point to allow enough time for fully opening the shutter. The shutter status is shown in brown in Fig. 6 ▸ with *T*_s_ being its opening/closing time. When the motor reaches the first position, the PandABox sends the first acquisition trigger of 9 ms to the Xspress 3 Mini (shown in blue in Fig. 6 ▸). As the motor motion continues, subsequent triggers are sent at each Bragg motor position corresponding to 1 eV energy change. For each trigger the exact Bragg angles are saved for the rising and falling edges, and the average energy value is calculated (*E*_1avg_ to *E*_100avg_ in Fig. 6 ▸) and assigned to the fluorescence counts from that trigger. By the end of the scan the program saves a two-dimensional array of 100 energy values and corresponding fluorescence counts.

In general, the PandABox is the main hardware component of the scanning procedure that monitors and verifies the synchronous motion of the motors as well as triggers the data acquisition at precise energy values. Having a correctly configured and cabled PandABox along with a synchronous scanning routine for multiple motors can serve as a base for implementing a similar scan at other beamlines or facilities.

### 3.3. Finalizing the scan

**Figure 7:**
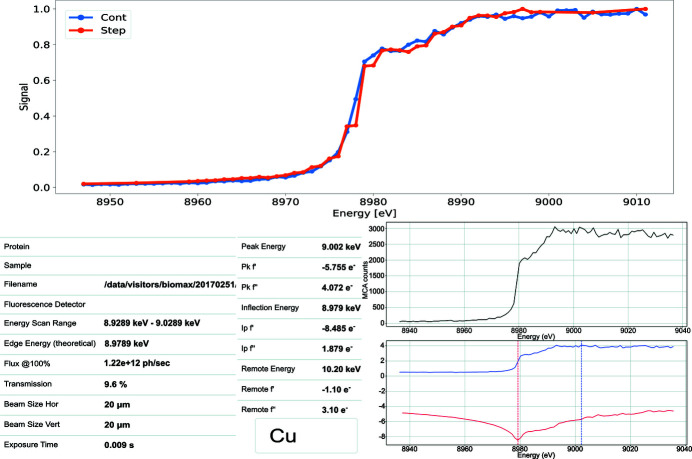
Top: comparison of continuous (blue) and step (orange) scans for the Cu *K*-edge. Bottom: screenshot of the energy scan results presented in *ISPyB* for the above scan.

Once the energy scan is completed, the output data are transferred to *CHOOCH* (Evans & Pettifer, 2001 ▸) – a data analysis program that computes the real and imaginary parts (*f* ′ and *f* *′′*, respectively) of the anomalous scattering factors as well as the inflection, peak and remote energy values. An example of a continuous scan around the Cu *K*-edge is shown in the top part of Fig. 7 ▸ (in blue), where it is also compared with a step scan (in orange) along the same energy range. One can see from the plot that the two scans match well with each other, resulting also in similar outcomes from the data processing. Upon the completion of the process, the users can view the resulting scan plot in *MXCuBE3* as well as in *ISPyB* (Delagenière *et al.*, 2011 ▸) – an information management system for the users of an MX beamline. Along with the scan plot, *ISPyB* displays also the data processing results as well as additional information about the element in question and beamline parameters. An example of such an *ISPyB* entry for the mentioned energy scan is shown in the bottom part of Fig. 7 ▸. The inflection, peak and remote energy values obtained from the scan are saved in the system and presented in *MXCuBE3* to the users as an option for the energy of the following data collection.

At the completion of the energy scan procedure the beam transmission and the photon energy are changed back to the original values before the start of the scan.

### 3.4. Duration of the scan

As stated earlier, the duration of the continuous scan itself is only 1 s, which is two orders of magnitude faster compared with a conventional step scan for the same energy range. The overall duration of the whole continuous scan routine, however, can vary largely depending on different initial conditions prior to the start of the scan. For example, depending on the original beamline energy before the scan, it can take from several seconds to a few minutes to move the energy to the starting position of the scan. The same amount of time will be spent after the completion of the scan to move the energy back to the original value. As discussed in Section 3.1 the energy change is usually followed by a *checkbeam* routine that waits for the beam to be stable and well aligned. Similar to the energy change, this procedure can also have different durations depending on the beam misalignment due to the energy movement and the convergence speed of the PID feedback loop under specific conditions. The energy changes and the subsequent beam alignment constitute the largest portion of the total duration and can add up to from a few tens of seconds to a few minutes. Similarly, the change of transmission from the original value to the nominal 0.5% and back as well as possible transmission adjustment described in Section 3.1 may require from a few seconds to a few tens of seconds. Other processes such as pre- and post-scan motion of the motors, reading/writing the data files, automatic data processing *etc*. are relatively fast and usually amount to around 10 s. From various scans performed at BioMAX the average total duration of the scan is about 80 to 90 s. Compared with the conventional step scan previously available at BioMAX, the newly implemented continuous scan is a factor of four to five faster.

## 4. Discussion

When discussing the performance and reliability of the energy scan it is important to take into consideration different aspects such as the synchronization of the motor motions, accuracy of the energy change, beam alignment during the scan and the robustness of data acquisition. For the latter, a 100 Hz acquisition is far below the upper limit of the Xspress 3 Mini device and so far no data losses or missed points have been observed during the measurements.

### 4.1. Motion linearity

**Figure 8:**
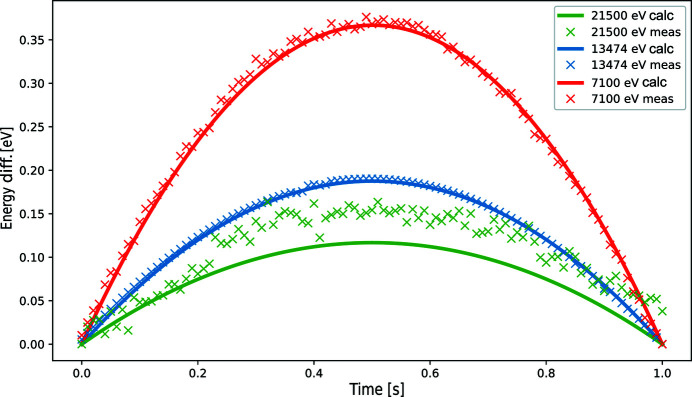
Difference between a linear energy change and a linear Bragg angle change. Solid lines correspond to calculated values while crosses represent measurements.

As mentioned earlier, the photon energy dependence on the Bragg angle is not linear (Fig. 3 ▸); however, for a small energy range a linearity can be assumed. Figure 8 ▸ shows the energy deviation from a linear change when moving the Bragg angle with a constant speed: θ(*t*) = θ_0_ + *V*_0_ *t* with θ_0_ being the Bragg angle at the start of the scan and *V*_0_ the constant speed. The solid lines and the crosses in the figure correspond to, respectively, calculated and measured values at different *E*_edge_ energies.

When changing the energy with a constant speed, the Bragg angle speed *V*_θ_(*t*) changes in time, always decreasing towards higher energies (seen also in Fig. 3 ▸). During the scan, however, a constant Bragg angle speed *V*_0_ is used as an average of *V*_θ_(*t*) along the scan range. This is why the offsets in Fig. 8 ▸ increase while *V*_θ_(*t*) > *V*_0_ and start decreasing from about the mid-point on when *V*_θ_(*t*) < *V*_0_. One can see from the figure that the maximum deviation is less than 1 eV and it is smaller at higher energies. The latter can be deduced from Fig. 3 ▸, where the blue curve becomes more linear towards higher energies. The same curve also shows that the energy is more sensitive to the Bragg angle changes at higher energies. This fact was observed during the measurements where small deviations in the Bragg motor motion correspond to larger energy fluctuations and offsets as shown in Fig. 8 ▸ (green crosses). The non-linear energy change during the scan is compensated by the fact that the PandABox triggers data acquisitions based on the Bragg angle values (and not time steps), and the corresponding energy values (which can also be non-equidistant) are assigned to the acquisitions.

### 4.2. Synchronization and stability

**Figure 9:**
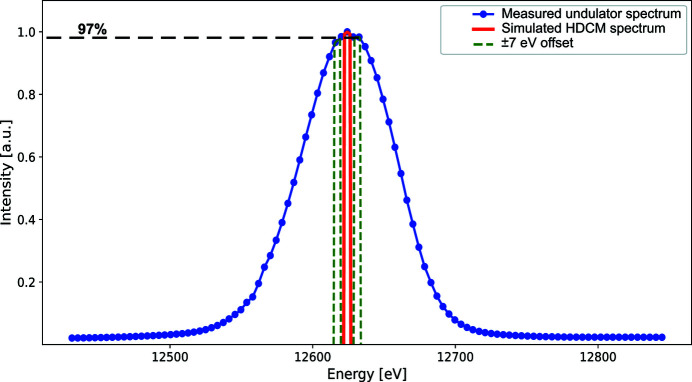
Comparison of the HDCM spectral bandwidth (red) and the undulator radiation spectrum (blue) at the seventh harmonic. Green curves correspond to ±7 eV offset from the centre of undulator spectrum.

As discussed in Section 3.2, the undulator gap and the Bragg motors are moving synchronously during the scan to continuously change the photon energy at the sample position. The final energy is defined by the Bragg angle of the monochromator that chooses a narrow energy bandwidth of 2 × 10^−4^ from a broader energy spectrum of undulator radiation. This is illustrated in Fig. 9 ▸ where the measured undulator spectrum (blue curve) and simulated HDCM spectrum (red curve) are compared. By synchronizing the motion of the two above-mentioned motors, we ensure that the HDCM narrow spectrum remains in the centre of the undulator spectrum as they both move from *E*_edge_ − 50 eV to *E*_edge_ + 50 eV, thus keeping the resulting radiation flux unchanged throughout the scan.

**Figure 10:**
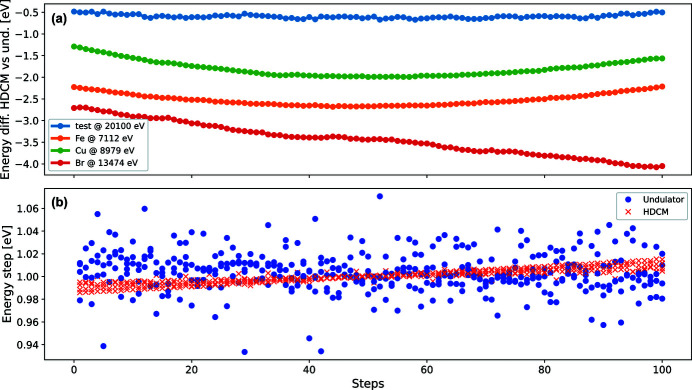
(*a*) Difference of the energy values for HDCM and undulator for four different scans. (*b*) Energy change per acquisition for HDCM (red crosses) and undulator (blue dots) plotted on top of each other for the same four scans.

As the final photon energy is defined by the Bragg angle and this value is used by the PandABox for energy calculation throughout the scan, any jitter or drift between the HDCM and undulator spectra does not result in uncertainty of the energy values of the scan. Instead, it affects the beam intensity resulting in fewer photons passing through the HDCM if its central energy has an offset from that of the undulator spectrum. For example, the green dashed curves in Fig. 9 ▸ correspond to an HDCM energy offset of ±7 eV which results in a drop of intensity to about 97%. These values were calculated for the seventh harmonic of the undulator; however, similar results can be obtained for all the other harmonics used at BioMAX. During the testing of the energy scan procedure a number of different parameters were captured and saved by the PandABox in addition to the fluorescence counts and HDCM energy values. Among these parameters were the energy values corresponding to the undulator gap during the scan and the resulting energy change per acquisition. The difference of the energy values corresponding to the scanning Bragg angles and undulator gaps is shown in Fig. 10 ▸(*a*), where the four curves correspond to scans at four different energies.

The figure shows that the largest energy difference observed from the test measurements is about 4 eV (illustrated by the red curve), which according to Fig. 9 ▸ corresponds to only a few percent intensity drop. Such a small drop is negligible for the energy scan application and does not affect the final results of the scan. It is worth noting that the largest deviation between the HDCM and undulator energies was observed during the scan at 13474 eV (around the Br *K*-edge). This is explained by the fact that at this energy the undulator gap is very small – about 5.25 mm (as seen in Fig. 3 ▸) – resulting in very strong magnetic fields inside the undulator gap, which implies a high mechanical load on the motors moving the gap and, consequently, less reliable gap movement during the scan. Figure 10 ▸(*b*) demonstrates the energy change per acquisition during the scan for the Bragg and undulator gap motor motion. The values for four different scans [same as in Fig. 10 ▸(*a*)] are plotted on top of each other. One can see from the figure that the distribution of the energy change values is well centred around 1 eV for all the steps with a maximum deviation of about ±0.06 eV. This proves that the energy change during the scans is fairly linear and reproducible.

### 4.3. Beam alignment

Another important consideration during the energy scan is the beam alignment. Ideally, the beam centring at the sample position should not be affected by a small energy change when the undulator and monochromator energies are changed synchronously. However, because of non-perfect synchronization of the undulator gap and Bragg motors (Fig. 10 ▸) as well as a possible overall misalignment of the beamline (caused by incorrect pitch and roll values of the HDCM second crystal and the focusing mirrors) this effect can become significant. Since the beam passes through various slits and apertures before reaching the sample position, any misalignments will result in partial cutting of the beam and, therefore, reduction of the beam intensity at the sample position. The beam centring PID feedback loop available at BioMAX is too slow to react to possible beam position shifts happening in a millisecond time scale, and the applied corrections might actually misplace the beam even further rather than keeping it centred throughout the scan. For this reason it is disabled just before the start of the scan and is re-enabled upon its completion.

**Figure 11:**
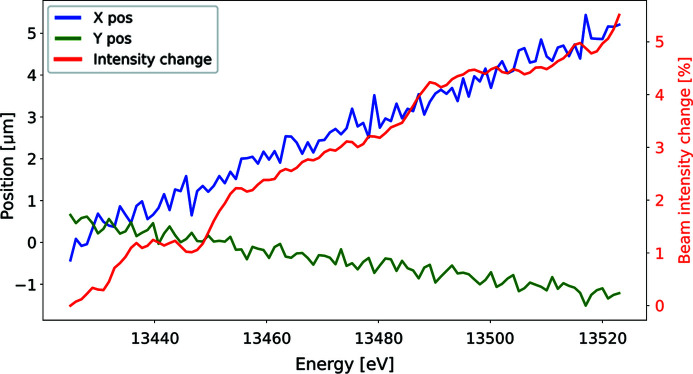
Beam position and intensity changes during the energy scan.

In order to monitor the beam alignment changes during the scan, test measurements were performed using an XBPM in the BCU (discussed in Section 2.1) and a fast photodiode close to the sample position for measuring the beam position and intensity, respectively. An example of such measurements for a scan around the Br *K*-edge is shown in Fig. 11 ▸. One can see from the figure that the beam position in the horizontal plane (blue curve) is shifted by about 5 µm while in the vertical (green curve) the shift is less than 2 µm.

This is explained by the horizontal geometry of the monochromator, meaning that the beam alignment in the horizontal plane is more sensitive to energy changes than in the vertical plane. The figure also shows the relative intensity drop, indicated by the red curve, which reaches the level of 5% and is consistent with the discussed position change. Such intensity changes do not affect significantly the fluorescence counts during the scan, and the outcome of the data processing is not distorted by them. Similar results were also observed for the scans at other energies, as far as the beamline itself is well aligned prior to the energy scan. It is also worth noting that the fast undulator gap changes may affect the alignment of the electron beam in the storage ring thus disturbing the operation of other beamlines. In order to better understand and estimate the extent of this effect, special measurements were performed where the BioMAX undulator gap was moved at various speeds within the range required for the energy scan, while the photon beam alignment at other beamlines was monitored. The measurements showed that the fast orbit feedback (FOFB) system (Sjöström *et al.*, 2011 ▸) operating at the storage ring was able to compensate any electron beam disturbances caused by the BioMAX undulator motion, and while FOFB was running no alignment changes were observed at other beamlines. However, when repeating the measurements without the FOFB running, some beam fluctuations were observed at other beamlines. It is, therefore, a pre­requisite to have the FOFB running at the storage ring to be able to perform the continuous energy scans at BioMAX.

## 5. Summary

A continuous energy scan routine has been developed and implemented at the BioMAX beamline for measuring the fluorescence signal near the absorption edges of heavy elements present in macromolecules. The scanning duration is as short as 1 s making it two orders of magnitude faster compared with the conventional step scan. Owing to this continuous scanning as well as other improvements in the preparation and completion phases of the routine, its overall duration is reduced by a factor of four to five. Furthermore, the motion trajectories and the synchronization of the motors as well as the stability of the process were investigated proving that it can be reliably utilized during the user operation. The implementation of the continuous energy scan is an important upgrade of the BioMAX beamline and provides the users with a fast and easy access to anomalous diffraction data from their samples. This, in turn, simplifies the structure reconstruction with experimental phasing using the SAD or MAD techniques. The method used for the continuous energy scan is not specific solely to the BioMAX beamline and can be implemented at other beamlines.
